# Upregulation of brain-derived neurotrophic factor by Shiikuwasha (*Citrus depressa* Hayata)

**DOI:** 10.1186/s40780-023-00309-7

**Published:** 2023-12-12

**Authors:** Kensuke Nakajima, Shinya Okubo, Tomoe Ohta, Takuhiro Uto, Shigeru Oiso

**Affiliations:** 1 Department of Pharmacy, Faculty of Pharmaceutical Sciences, Nagasaki International University, 2825-7 Huis Ten Bosch, Sasebo, Nagasaki 859-3298 Japan; 2 Graduate School of Pharmaceutical Sciences, Nagasaki International University, 2825-7 Huis Ten Bosch, Sasebo, Nagasaki 859-3298 Japan

**Keywords:** Depression, Brain-derived neurotrophic factor, ACHN cell line, Shiikuwasha

## Abstract

**Background:**

A reduction in the brain-derived neurotrophic factor (BDNF) level in the brain causes depression, whereas an increase in its level has therapeutic benefits against depression. BDNF is synthesized in various peripheral tissues and transported to the brain via the peripheral circulation across the blood–brain barrier. Therefore, substances that upregulate peripheral BDNF level may be used to prevent and treat depression. Previously, we demonstrated that *Citrus unshiu* peel (Chinpi) and *C. natsudaidai* increased BDNF level in a human renal adenocarcinoma cell line ACHN, which has BDNF-producing ability. Here, we evaluated whether Shiikuwasha (*C. depressa* Hayata), a citrus species cultivated in East Asia, can upregulate BDNF level in ACHN cells.

**Methods:**

We evaluated the effects of test samples on BDNF production by measuring BDNF level in the medium of ACHN cells after a 24 h cultivation in the presence of test samples. The *BDNF* mRNA level was measured by quantitative reverse transcription-polymerase chain reaction, and the phosphorylation level of cyclic adenosine monophosphate response element-binding protein (CREB), a transcription factor regulating BDNF expression, was determined using Western blotting.

**Results:**

We found that methanol extracts of Shiikuwasha peel, pulp, and seed increased the BDNF level in the culture medium of ACHN cells. Shiikuwasha peel and pulp extracts also upregulated *BDNF* mRNA level and phosphorylation of CREB.

**Conclusions:**

These results suggest that Shiikuwasha includes the candidate antidepressant substances with peripheral BDNF-upregulation effect.

## Background

Depression is a common mental disorder characterized by low mood, loss of interest, anhedonia, sleep disturbance, and suicidal tendencies and negatively affects human well-being and socio-economic aspects [1, 2]. Currently, clinically available antidepressants, such as selective serotonin reuptake inhibitors, serotonin-noradrenaline reuptake inhibitors, and tricyclic and tetracyclic antidepressants, are based on the hypothesis that monoamine deficiency in the brain causes depression. However, these drugs are ineffective in approximately 30% of patients with depression [3] and take several weeks to elicit an antidepressant effect [4]. Additionally, there are no effective prophylactic strategies against depression. Therefore, novel therapeutic and prophylactic approaches for depression are necessary.

Brain-derived neurotrophic factor (BDNF), first isolated from a pig brain in 1982 [5], is a critical neurotrophin for neurogenesis, neuronal differentiation, neuroprotection, and synaptic plasticity in the central nervous system (CNS) [6, 7]. BDNF is a 14-kDa protein composed of 119 amino acids. *BDNF* transcription is regulated by the cyclic adenosine monophosphate (cAMP) response element-binding protein (CREB) signaling pathway [8, 9]. CREB is activated by phosphorylation at Ser-133 [10]. CREB phosphorylation is regulated by the activation of various signaling pathways, including phosphoinositide 3-kinase/protein kinase B (Akt), cAMP-dependent protein kinase (PKA), calcium/calmodulin-dependent protein kinase, and Ras/Raf/mitogen-activated protein kinase kinase (MEK)/extracellular signal-regulated kinase [9, 11, 12].

Reduction in BDNF level in the hippocampus is reportedly associated with depression [13, 14], and the BDNF level is reduced in the postmortem brains of patients with depression [15]. Moreover, the BDNF level in the hippocampus is reduced by various stresses such as chronic unpredictable mild stress, restraint stress, corticosterone injection, olfactory bulbectomy, and maternal stress in rodents [16–20], and BDNF administration to the hippocampus elicits antidepressant-like effects in rats [21, 22]. These results support the neurotrophic hypothesis of depression, that is, BDNF is a crucial factor in depression development [14]. Hence, BDNF upregulation in the brain is considered beneficial for preventing and treating depression.

BDNF is expressed in the CNS and is present in nearly all regions of the brain [23, 24], especially in the hippocampus and cerebral cortex. Additionally, BDNF is produced in the skeletal muscles, kidney, thymus, heart, liver, lung, and spleen [25–30]. It can be transported to the brain from the peripheral circulation by crossing the blood–brain barrier [31, 32]. Furthermore, a positive correlation exists between BDNF level in the brain and that in the blood [33], and peripheral BDNF administration exerts an antidepressant-like effect and upregulates BDNF level in the hippocampus [34]. Therefore, compounds that elevate BDNF level in the peripheral circulation potentially exhibit prophylactic and therapeutic effects against depression due to an increase in the level of BDNF transported to the brain.

In our previous study, we demonstrated that the human renal adenocarcinoma cell line ACHN produces and secretes BDNF. Using the cell line, we screened for substances that upregulate BDNF level to identify prophylactic and therapeutic agents against depression [35]. We demonstrated that foxtail millet, some Kampo medicines (Japanese traditional medicines), such as Chotosan, Hochuekkito, Kososan, and Ninjinyoeito, and *Citrus unshiu* peel (crude drug; Chinpi), an ingredient of these four Kampo medicines, significantly increase BDNF level in the culture medium of ACHN cells [36, 37]. Moreover, we reported that *Citrus natsudaidai* upregulates BDNF protein and mRNA level in ACHN cells [38]. As treatment with *C. unshiu* and *C. natsudaidai* increased the BDNF level in the culture medium of ACHN cells [36, 38], *Citrus* species are thought to enhance BDNF production in ACHN cells.

Shiikuwasha (*Citrus depressa* Hayata) is a well-known *Citrus* species that grows natively in Taiwan, Korea, and Japan (Okinawa) [39]. Approximately 3.0 × 10^6^ kg of Shiikuwasha is produced each year in Okinawa and is used as a fresh fruit as well as for making juice, food additives, and functional foods. Shiikuwasha extract has anti-obesity effects and alleviates skeletal muscle atrophy and osteoarthritis progression [40, 41], however, it is unknown whether Shiikuwasha affects BDNF production. The aim of this study was to evaluate the effect of Shiikuwasha on BDNF level in ACHN cells.

## Methods

### Reagents and materials

Shiikuwasha (*Citrus depressa* Hayata) was supplied by the Division of Citrus Research, Institute of Fruit Tree and Tea Science, National Agriculture and Food Research Organization (NARO) (Shizuoka, Japan). They were harvested at NARO's farm on December 2, 2021. Botanical classification was conducted by NARO and Dr. Kensuke Nakajima. Voucher specimen of this plant (NO-Shii-001) is deposited at the Department of Pharmacy, Faculty of Pharmaceutical Sciences, Nagasaki International University (Nagasaki, Japan). The Quantikine® human BDNF Immunoassay kit (Catalog # DBD00) was purchased from R&D Systems (Minneapolis, MN, USA). Dulbecco’s modified Eagle’s medium (DMEM) and TRIzol were purchased from Life Technologies (Carlsbad, CA, USA), and ReverTra Ace was purchased from Toyobo (Osaka, Japan). TB Green Fast qPCR Mix was purchased from Takara Bio. Inc. (Shiga, Japan). The primers were obtained from GeneNet (Fukuoka, Japan) or FASMAC (Kanagawa, Japan). Antibody for CREB and pCREB (Ser-133), and RIPA lysis buffer were purchased from Cell Signaling Technology (Danvers, MA, USA). Anti-β-actin antibody was obtained from Sigma-Aldrich Co. (St. Louis, MO, USA). Hesperidin and nobiletin were purchased from Tokyo Chemical Industry Co. (Tokyo, Japan). 3-(4,5-Dimethyl-2-thiazolyl)-2,5-diphenyl-2*H*-tetrazolium bromide (MTT), dimethyl sulfoxide (DMSO), and other reagents were purchased from FUJIFILM Wako Pure Chemical Corporation (Osaka, Japan).

### Cell culture

Human renal adenocarcinoma ACHN cells were cultured in DMEM supplemented with 10% fetal bovine serum at 37 °C in a humidified atmosphere with 5% CO_2_.

### Preparation of Shiikuwasha extracts

Shiikuwasha was separated into peel (albedo and flavedo), pulp, and seeds. The peel and pulp were lyophilized using a freeze drier (FDU-1200, EYELA, Tokyo, Japan). The seeds were dried at room temperature for 48 h. Lyophilized peel and pulp, and dried seeds were crushed using a Tube Mill 100 control (IKA, Staufen, Germany). The crushed samples (5 g) were agitated in methanol for 12 h at 25 ± 2 °C. The supernatant was collected after centrifugation at 15,000 × *g* for 5 min and dried by spraying with nitrogen gas. Each residue was dissolved in DMSO at a final concentration of 40 mg/mL.

### MTT assay

An MTT assay was performed as described previously [36]. Briefly, the cells were seeded in 96-well plates at a density of 1 × 10^4^ cells/well and cultured for 24 h. Shiikuwasha extracts (0–100 μg/mL) were added to the culture medium. After 24 h of culture, MTT (200 μg/mL) was added to each well, and the cells were cultured for another 4 h. After removing the culture medium, formazan crystals were dissolved in DMSO. The optical density of the samples was measured at 570 nm using a Multiskan FC microplate reader (Thermo Fisher Scientific, Waltham, MA, USA). The values were expressed as the ratio of the optical density of test extracts-treated cells to that of the control cells.

### Measurement of BDNF level in the culture medium of ACHN cells

ACHN cells were seeded in 96-well plates at a density of 1 × 10^4^ cells/well and cultured for 24 h in DMEM. Fresh DMEM with or without Shiikuwasha extracts was added to each well, and the cells were cultured for 24 h. Thereafter, the culture medium was collected, and the level of BDNF in the culture medium was measured using the Quantikine® human BDNF Immunoassay kit, according to the manufacturer’s instructions.

### Quantitative reverse transcription-polymerase chain reaction (qRT-PCR)

The total RNA was extracted from ACHN cells treated with or without the test samples using TRIzol reagent and reverse-transcribed to complementary DNA (cDNA) using ReverTra Ace. The reaction mixture, containing 10 μL of 2 × TB Green Fast qPCR Mix, 0.8 pmol of sense and antisense primers, and 2 μL of diluted cDNA, was loaded into a 96-well plate. PCR amplification was conducted under the following conditions: 95 °C for 30 s, followed by 40 cycles at 95 °C for 5 s and 60 °C for 10 s. PCR was performed using the CFX Connect Real-Time System (Bio-Rad, Hercules, CA, USA). The following sense and antisense primers were used: human BDNF, forward 5′-TTTGGTTGCATGAAGGCTGC-3′ and reverse 5′-GCCGAACTTTCTGGTCCTCA-3′; 18S ribosomal RNA, forward 5′-GTAACCCGTTGAACCCCATT-3′ and reverse 5′-CCATCCAATCGGTAGTAGCG-3′. Transcript levels were estimated from the respective standard curves and normalized to 18S ribosomal RNA expression (internal control).

### Western blotting

ACHN cells were seeded in 6-cm dishes at a density of 0.8 × 10^6^ cells/dish. Twenty-four hours later, the cells were treated with methanol extracts of Shiikuwasha peel and pulp (50 and 100 μg/mL) for 24 h, after which they were harvested and lysed in RIPA lysis buffer containing protease and phosphatase inhibitors. The samples were centrifuged at 12,000 × *g* for 15 min at 4 °C, and the protein content of the samples was determined using a dye-binding protein assay kit according to the manufacturer’s instructions (Bio-Rad). Equal amounts of proteins were subjected to sodium dodecyl sulfate–polyacrylamide gel electrophoresis, transferred onto polyvinylidene fluoride membranes, and detected following the method reported by Uto et al. [42].

### HPLC analysis

The HPLC system was performed on a JASCO LC-2000 Plus series (JASCO, Tokyo, Japan). TSKgel ODS-100V column (4.6 × 250 mm, particle size 5 µm, Tosoh Corp. Tokyo, Japan) was used for HPLC analyses at 40 °C. The mobile phases 0.1% formic acid in acetonitrile and 0.1% formic acid in water eluted according to the following gradient program: 0–20 min (10:90 → 100:0, v/v) → 20–30 min (100:0, v/v) → 30–40 min (10:90, v/v). The flow rate was 1 mL/min, the injection volume was 10.0 µL, and the detection wavelength was 275 nm.

Sample and standard solutions were prepared by dissolving each DMSO solution in methanol followed by HPLC analysis. For the sample solutions, 40 mg/mL of the Shiikuwasha extracts (peel, pulp, and seeds) were diluted to a final concentration of 4.0 mg/mL. Hesperidin and nobiletin were used as standards. Hesperidin (50 µmol/L) and nobiletin (25 µmol/L), both diluted to 25 nmol/L separately, were used as standard solutions, and their peaks were obtained at 9.5 and 16.4 min, respectively.

### Statistical analysis

Values are presented as mean ± standard deviation. Differences between the groups were analyzed using the one-way analysis of variance, followed by Dunnett's test. Differences were considered significant at *P* < 0.05.

## Results

### Effect of the methanol extracts of Shiikuwasha on BDNF level in ACHN cells

If the number of ACHN cells is substantially increased with the Shiikuwasha treatment, then the level of BDNF in the culture medium will also increase regardless of the ability of ACHN cells to produce BDNF. There was no significant increase in the viability of ACHN cells treated with Shiikuwasha extract at 50 and 100 µg/mL compared with that of the control, as determined using the MTT assay (data not shown). Therefore, we investigated the effect on BDNF level in ACHN cells using 50 and 100 µg/mL of each Shiikuwasha extract.

**Fig. 1:**
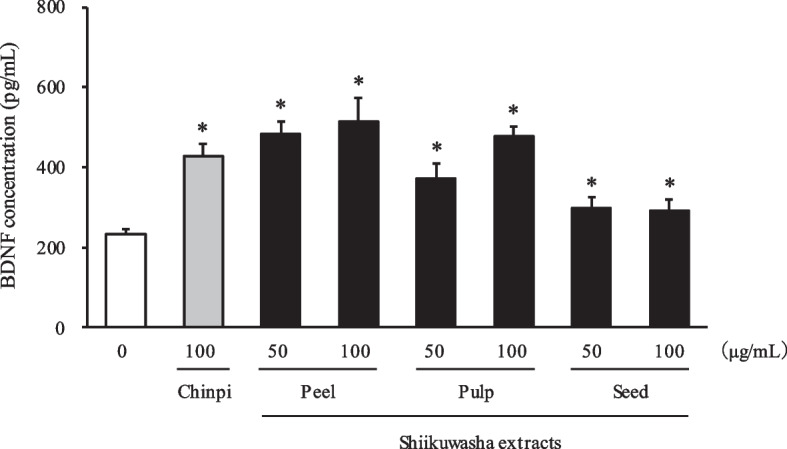
Effects of Shiikuwasha extracts on brain-derived neurotrophic factor (BDNF) level in ACHN cell culture medium. ACHN cells were cultured for 24 h with the methanol extracts of Shiikuwasha peel, pulp, and seeds at 50 and 100 µg/mL. BDNF level was measured using the enzyme-linked immunosorbent assay. Each value represents BDNF level in the culture medium. ACHN cells treated with Chinpi were used as the positive control. Data are expressed as mean ± SD (controls; *n* = 4, other groups; *n* = 3). Dunnett's test; *P < 0.05 vs. control (0 µg/mL)

Shiikuwasha peel extracts significantly increased the BDNF level in the culture medium, 2.08-fold increase (484.4 pg/mL) upon treatment with 50 μg/mL extract and 2.20-fold increase (513.9 pg/mL) upon treatment with 100 μg/mL extract, compared with that of the control (233.1 pg/mL) (Fig. 1). Shiikuwasha pulp extracts significantly increased the BDNF level in the culture medium, 1.60-fold increase (372.5 pg/mL) at 50 μg/mL and 2.05-fold increase (478.0 pg/mL) at 100 μg/mL, compared with that of the control (233.1 pg/mL) (Fig. 1). Furthermore, Shiikuwasha seed extracts significantly upregulated the BDNF level, 1.28-fold increase (298.3 pg/mL) at 50 μg/mL and 1.26-fold increase (292.7 pg/mL) at 100 μg/mL, compared with that of the control (233.1 pg/mL) (Fig. 1). The BDNF concentration in the culture medium treated with Chinpi (100 μg/mL; positive control), which was found to upregulate BDNF previously [36], was 429.1 pg/mL (Fig. 1).

### Effects of Shiikuwasha extracts on *BDNF* mRNA level in ACHN cells

**Fig. 2:**
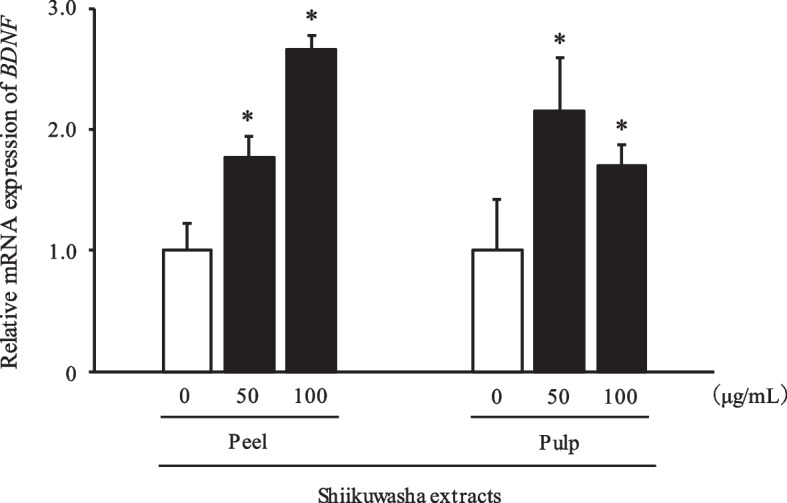
Effects of methanol extracts of Shiikuwasha peel or pulp on *BDNF* level in ACHN cells. ACHN cells were cultured for 24 h with each extract. *BDNF* level was measured using quantitative reverse transcription-polymerase chain reaction. Each value represents *BDNF* level of treated cells relative to that of the control (0 µg/mL). Data are expressed as mean ± SD (peel at 50 µg/mL; *n* = 3, other groups; *n* = 4). Dunnett's test; *P < 0.05 vs. control (0 µg/mL)

As Shiikuwasha peel and pulp methanol extracts strongly upregulated the BDNF level in ACHN cells, we further evaluated *BDNF* expression in ACHN cells treated with Shiikuwasha peel and pulp extracts using qRT-PCR. *BDNF* expression in ACHN cells treated with peel extract increased by 1.8-fold at 50 μg/mL and 2.7-fold at 100 μg/mL compared with that of the control (Fig. 2). In cells treated with pulp extract, *BDNF* expression increased by 2.2-fold at 50 μg/mL and 1.7-fold at 100 μg/mL compared with that in the control cells (Fig. 2).

### Effects of Shiikuwasha extracts on CREB phosphorylation in ACHN cells

**Fig. 3:**
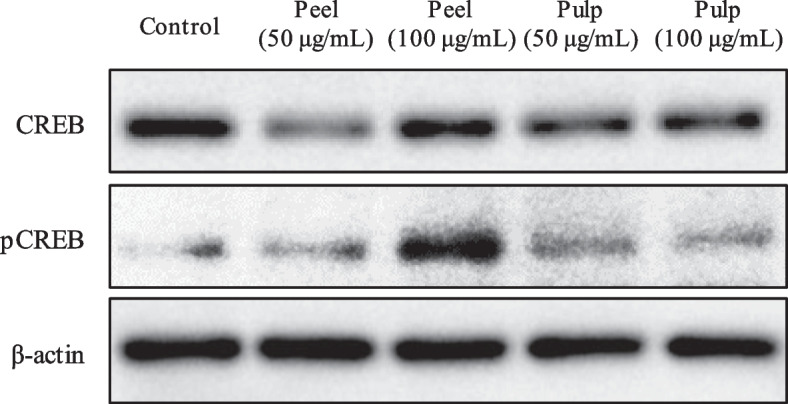
Effects of methanol extracts of Shiikuwasha peel or pulp on CREB phosphorylation in ACHN cells. ACHN cells were cultured for 24 h with the methanol extracts of Shiikuwasha peel and pulp at 50 and 100 µg/mL. CREB and pCREB expression was evaluated using Western blotting

*BDNF* transcription is regulated by phosphorylated CREB [8, 9]. As an increase in *BDNF* transcription by Shiikuwasha extracts was observed, we examined whether Shiikuwasha extracts affect CREB phosphorylation. Phosphorylated CREB expression in ACHN cells treated with Shiikuwasha peel and pulp extracts was higher than that of the control (Fig. 3).

### HPLC analysis of Shiikuwasha extracts

**Fig. 4:**
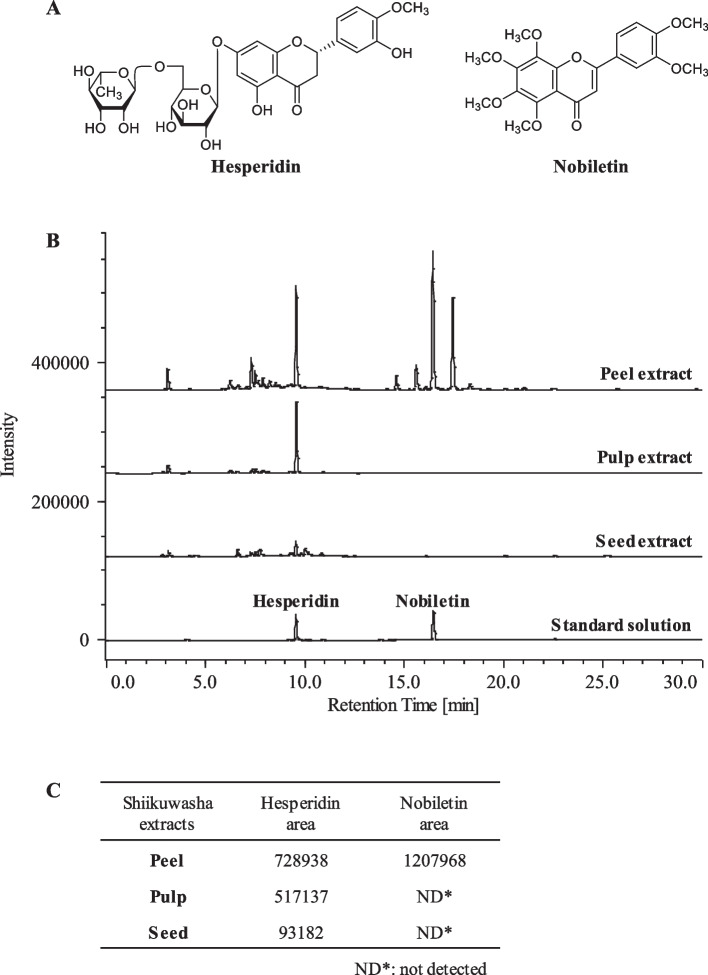
**A** Chemical structures of hesperidin and nobiletin. **B** HPLC chromatograms of Shiikuwasha extracts (peel, pulp, and seeds: 4.0 mg/mL) and standard solution (hesperidin and nobiletin: 25 nmol/L). Retention times of hesperidin and nobiletin were 9.5 and 16.4 min, respectively. **C** Peak areas of hesperidin and nobiletin from Shiikuwasha extracts

Shiikuwasha is particularly rich in flavonoids such as hesperidin and nobiletin (Fig. 4A) [43]. Hesperidin and nobiletin are reportedly present in higher concentrations in the Shiikuwasha peel than in the pulp [43]. To confirm that Shiikuwasha contains these compounds, each Shiikuwasha extract was subjected to HPLC analysis. Hesperidin was identified in all extracts by its peak at 9.5 min, corresponding to the retention time of the compound (Fig. 4B). Furthermore, the observed peak areas suggested higher content in the peel, pulp, and seed, in that order (Fig. 4C). Nobiletin was only identified in the peel extract by its peak at 16.4 min, corresponding to the retention time of the compound (Fig. 4B).

## Discussion

Based on the neurotrophic hypothesis of depression proposed by Duman et al. [14] and Duman and Monteggia [13], upregulation of BDNF level in the brain region can serve as an efficient prophylactic and therapeutic strategy against depression. Considering that BDNF crosses the blood–brain barrier [31] and BDNF administration peripherally induces an increase in BDNF level in the hippocampus [34], upregulation of peripheral BDNF level could increase brain BDNF level and exert an antidepressant effect.

Here, we demonstrated that the methanol extracts of Shiikuwasha (peel, pulp, and seeds) upregulated the BDNF level in the culture medium of ACHN cells. The BDNF-upregulating effects of peel and pulp were almost the same as those of Chinpi (positive control). Furthermore, Shiikuwasha peel and pulp extracts upregulated *BDNF* level and CREB phosphorylation. Although Shiikuwasha has an anti-obesity effect and other therapeutic properties [40, 41], no study has investigated its effect with respect to upregulating BDNF level and acting as an antidepressant. Our results indicate that Shiikuwasha can be a candidate antidepressant food with a peripheral BDNF-upregulation effect.

Nogata et al. reported that Shiikuwasha contains hesperidin (a type of flavanones) and nobiletin (a type of flavones) [43]. HPLC analysis revealed that hesperidin was rich in the peel and pulp of Shiikuwasha extracts used in this study. Hesperidin reportedly has BDNF-upregulating effects [44] and activates the PKA/CREB/BDNF pathway in vivo [45]. Therefore, hesperidin is likely related to Shiikuwasha peel and pulp-induced BDNF upregulation. HPLC analysis confirmed the presence of nobiletin in peel extracts but not pulp and seed extracts. Nobiletin exerts a BDNF-increasing effect in stressed rats hippocampus [46]. Nobiletin may have partially contributed to the BDNF-increasing effect of Shiikuwasha peel. Hesperidin and nobiletin, which are abundant in Shiikuwasha, reportedly exerted antidepressant effects and attenuated the reduction in hippocampal BDNF level in a mild traumatic brain injury- and chronic mild stress-induced rodent model of depression, respectively [44, 46]. Furthermore, consumption of flavonoids (such as hesperidin, narirutin, and nobiletin)-rich orange juice increases the serum BDNF level in young adults with depressive symptoms [47]. Therefore, Shiikuwasha, a citrus fruit rich in hesperidin and nobiletin, is thought to be beneficial in preventing and treating depression.

The development of therapeutic agents for CNS disorders, such as depression, Alzheimer’s disease, and Parkinson’s disease, has not been as successful as that for peripheral diseases, owing to low permeability of compounds into the brain [48]. Therefore, it is important to find agents that can be clinically used without considering their brain penetration. Here, we demonstrated that Shiikuwasha extracts increased the BDNF level in human renal adenocarcinoma ACHN. Schmidt and Duman reported that subcutaneous BDNF administration (8 µg/day for 14 d) increased the level of BDNF in the mouse hippocampus compared with that in the control [34], suggesting that peripheral BDNF upregulation leads to an increase in brain BDNF level. As our results were from an in vitro analysis, we cannot definitively conclude that Shiikuwasha increases the peripheral BDNF level in vivo. However, we previously demonstrated that red foxtail millet eliciting an approximately 1.4-fold increase in BDNF level in ACHN cells could increase rat serum BDNF level by approximately 1.3-fold [37]; in particular, the BDNF level in the culture medium treated with 100 μg/mL red foxtail millet and control were 250.0 pg/mL and 175.2 pg/mL, respectively. The peel and pulp of Shiikuwasha showed a greater BDNF-upregulating effect than that of red foxtail millet in ACHN cells; therefore, Shiikuwasha can potentially increase BDNF level in vivo. If Shiikuwasha increases the serum BDNF level, then the BDNF level in the brain will increase with the transport of peripheral BDNF through the blood–brain barrier.

The serum BDNF level in healthy individuals is approximately 1.2-fold higher than that in patients with depression [49]. Moreover, even among healthy individuals, the serum BDNF level is lower in individuals with occupational stress than in individuals who are not regularly exposed to stress [50]. Considering that Shiikuwasha peel and pulp extracts elicited an approximately twofold higher effect on ACHN cells, and BDNF is largely synthesized in the kidney [29], Shiikuwasha may be useful as a prophylactic and therapeutic agent against depression. However, the effect of the in vivo treatment of Shiikuwasha on peripheral BDNF level remains unclear. In the future, we plan to investigate whether the administration of Shiikuwasha upregulates BDNF level and induces antidepressant-like effects in a rat model of depression.

## Conclusions

We demonstrated the BDNF-upregulating effect of the methanol extracts of Shiikuwasha peel, pulp, and seeds in the culture medium of ACHN cells. Furthermore, we revealed that the extracts of Shiikuwasha peel and pulp upregulate *BDNF* expression. Hence, our study reports the novel function of Shiikuwasha as potential agent for the prevention and treatment of depression. Further studies are required to identify the active compounds demonstrating BDNF upregulation and to reveal that Shiikuwasha and those active compounds induce antidepressant-like effects in vivo.

## Data Availability

The datasets during and/or analysed during the current study available from the corresponding author on reasonable request.

## Abbreviations

Akt: Protein kinase B
BDNF: Brain-derived neurotrophic factor
cAMP: Cyclic adenosine monophosphate
cDNA: Complementary DNA
CNS: Central nervous system
CREB: Cyclic adenosine monophosphate response element-binding protein
DMEM: Dulbecco’s modified Eagle’s medium
DMSO: Dimethyl sulfoxide
MTT: 3-(4,5-Dimethyl-2-thiazolyl)-2,5-diphenyl-2*H*-tetrazolium bromide
MEK: Mitogen-activated protein kinase kinase
NARO: National Agriculture and Food Research Organization
PKA: Cyclic adenosine monophosphate-dependent protein kinase
qRT-PCR: Quantitative reverse transcription-polymerase chain reaction
